# Writing, Reading, and Listening Differentially Overload Working Memory Performance Across the Serial Position Curve

**DOI:** 10.5709/acp-0179-6

**Published:** 2015-12-31

**Authors:** Richard Tindle, Mitchell G. Longstaff

**Affiliations:** Health and Human Sciences, Southern Cross University, Coffs Harbour, New South Wales, Australia

**Keywords:** working memory, reading, listening, writing, serial recall

## Abstract

Previous research has assumed that writing is a cognitively complex task, but has not determined if writing overloads Working Memory more than reading and listening. To investigate this, participants completed three recall tasks. These were reading lists of words before recalling them, hearing lists of words before recalling them, and hearing lists of words and writing them as they heard them, then recalling them. The experiment involved serial recall of lists of 6 words. The hypothesis that fewer words would be recalled overall when writing was supported. Post-hoc analysis revealed the same pattern of results at individual serial positions (1 to 3). However, there was no difference between the three conditions at serial position 4, or between listening and writing at positions 5 and 6 which were both greater than recall in the reading condition. This suggests writing overloads working memory more than reading and listening, particularly in the early serial positions. The results show that writing interferes with working memory processes and so is not recommended when the goal is to immediately recall information.

## Introduction

Working Memory (WM) is a limited capacity system devoted to the temporary storage, retrieval, and manipulation of information during a variety of cognitive processes (Baddeley, 2000; Baddeley & Hitch, 1974). WM also plays a significant role in our ability to process and perform complex cognitive tasks such as listening, reading, and writing (Olive, 2004; Tirre & Peńa, 1992). Baddeley (2003, 2012) maintains that WM is comprised of four systems: The central executive is responsible for devoting attentional processes to three sub-systems. The first sub-system is the phonological loop, which is responsible for the temporary storage of verbal information (written or spoken). The visuo-spatial sketchpad is responsible for temporarily storing visual and spatial information such as colour, speed, shape, and movement. The final sub-system is the episodic buffer (Baddeley, 2000), which is controlled by the central executive and is able to process and integrate multi-coded information (phonological, visual, spatial, and long-term memory).

The multi-coded nature of WM enables the integration of information from the phonological loop, visuo-spatial sketchpad, and long-term memory to aid in problem-solving (Baddeley, 2000, 2001). Baddeley (2012) also explains that the central executive plays a prominent role in directing attentional resources to the phonological loop, suggesting both systems play a key role in learning verbal and written information. When processing verbal information, the phonological loop is able to store phonologically coded information directly into temporary storage. However, due to WM’s limited available capacity it can become overloaded during cognitively complex tasks (McCutchen, 1996, 2000; Olive, 2004; Peverly, 2006; Schweppe & Rummer, 2013).

Attention is also a limited capacity resource that is integral to WM processes such as encoding and maintenance (Barrouillet & Camos, 2007; Chun, 2011). Chun investigated the relationship between visual WM and visual attention. Despite identifying that both of these operate independently and are limited in capacity, the performance of the tasks was determined by how well distractions could be inhibited. The ability to sustain and direct attention towards relevant items is important for successful WM performance when faced with both internal and external distractors. Furthermore, when performing WM tasks, we switch our attention and resources between the encoding and maintenance of to-be-remembered information, as explained by the time-based resource-sharing model (Barrouillet & Camos, 2007). According to this model, if attentional resources are diverted away from one process (e.g., encoding the words), they cannot be effectively used for that process as they are now being used for the process they are diverted to (e.g., maintenance). Barrouillet and Camos also demonstrated that there is a greater decline in WM performance the longer a secondary process captures attention. This suggests that when attention is captured by a secondary task it can prevent attention from being directed towards WM encoding and maintenance. If the secondary task can be inhibited and/or does not substantially capture attention, WM performance will be more successful. The aim of the current study is to investigate how the relative complexity and processing demands of listening, reading, and writing tasks affect our ability to recall information.

Listening is a relatively simple task that places little strain on WM when required to process, rehearse, and retrieve information within the phonological loop (Christensen et al., 2012; Margolin, Griebel, & Wolford, 1982). When verbal information is heard (e.g., while listening to speech) it can be stored directly into the phonological loop as it is phonologically coded (Baddeley & Larsen, 2007; Haenggi & Perfetti, 1992). However, WM is still limited in its capacity to store information and not all verbal information enters the phonological loop (Chen & Cowan, 2009). This may occur when the central executive must divide attention between two cognitive processes, for example, when there are dual tasks (Barrouillet & Camos, 2007; Unsworth & Engle, 2007). It can also occur if a distraction interrupts sub-vocal rehearsal such as when two verbal tasks occur simultaneously (e.g., speaking while listening to a conversation), resulting in articulatory suppression (Chen & Cowan, 2009; Oberauer & Lewandowsky, 2008). The addition of factors such as articulatory suppression and dividing attention may contribute to overloading WM capacity, resulting in poor verbal WM performance. Given the above, listening by itself appears to be a relatively simple-task. Other verbal tasks such as reading also utilise the phonological loop but seem to be relatively more complex.

The cognitive processes involved during reading appear to be more complex than those occurring during a listening task (Margolin et al., 1982; Rayner, Pollatsek, Ashby, & Clifton, 2012). When reading, information must be converted into a phonological code before being temporarily stored in the phonological loop (Baddeley, 1997; Sadoski & Paivio, 2004). This is achieved through the articulatory control process by sub-vocalising the written material (Lewandowsky & Farrell, 2006; Page & Norris, 1998; Tan & Ward, 2008). This creates an additional step for the reading process before the words can be temporarily stored, which places greater strain on WM (Davidson, 1986). This increase in complexity may be due to the phonological loop’s inability to transform written material into a phonological code efficiently during complex tasks (Besner & Davelaar, 1982; Folk, 1999). Research has shown that cognitive resources devoted to reading can overload WM’s capacity to store and immediately recall information (Linderholm, Xiaosi, & Qin, 2008; McCutchen, 2000; Olive, 2004; Peverly, 2006). In contrast to listening, the reading process appears to be relatively more complex due to the additional transformation of the words into a phonological code. Complexity can also be increased in other ways, such as when required to write down information while listening, raising the demands on WM processes.

When listening to verbal information that we might want to later recall (e.g., during a lecture) it is common to write down key points as we hear them. However, the production of written material places significant cognitive demands on WM and can hinder the recall of to-be-remembered information (Bourdin & Fayol, 1994; Kellogg, 1996, 2001; Klein & Boals, 2001; McCutchen, 1996, 2000; Olive, 2004; Peverly, 2006). Our ability to store information in WM seems to be impaired when an individual is asked to write down information while listening (e.g., Bourdin & Fayol, 1994; McCutchen, 1996; Peverly, 2006). Peverly attributes this to the complexity of the writing process, as it requires a high level of cognitive effort. This places extra strain on WM, inhibiting its processes and our ability to store information (McCutchen, 2000). Writing is a cognitively complex task requiring greater effort, overloading WM and its capacity to store verbal information as well as devote processes to writing. However, these studies have not identified if the relative complexity of writing reduces WM performance compared to just listening to information or reading information when information must be immediately recalled.

Kellogg (1996) produced a model of writing and WM showing the interrelation between the two processes to identify that they share a common resource. Kellogg suggested writing loads on WM processes because we must plan, execute, and monitor written output. When planning to write, verbal WM is activated to plan the phonetics of the words (e.g., spelling, letters, sounds, and syllables). In addition to this, the movement requirements are planned within visuo-spatial WM to produce legible letter shapes and maintain the correct spatial sequence of letters. After the planning stage, the written output is executed and is the responsibility of the central executive (Kellogg, 1996). During execution, constant visual feedback is required to edit and maintain the written output to ensure what has been written and what comes next will be correct (Kellogg, 1996). Any errors or perceived errors are rectified and adjustments in motor movements and/or the phonetics of the words are made. Recent research has further demonstrated that handwriting is a complex motor task that requires visual feedback to be executed efficiently (Tse, Thanapalan, & Chan, 2014). Kellogg’s model showed that writing and WM share a common resource as well as identifying how the different writing processes utilise verbal, visual, spatial, and executive processes within WM.

The processes involved in writing appear to overload WM’s ability to devote resources to both writing and information storage (Kellogg, 1996; McCutchen, 2000; Peverly, 2006). McCutchen suggests that due to the cognitive complexity of the writing process and the limited capacity of WM, trade-offs exist between task fluency (e.g., speed of writing) and information storage and retrieval. For example, when participants devoted attentional resources to the writing movement (as defined by the speed of letter production) their ability to store relevant information was adversely affected. However, when participants devoted attention to the storage of information, the fluency of their writing was hindered. This switching and trading off of resources is controlled by the central executive. Kellogg suggested that the central executive also plays a significant role in processing cognitive information during difficult tasks, such as writing.

Kellogg (1996) argues that the central executive may be impaired when higher-level cognitive demands are placed on it. For example, during complex tasks such as writing, it is unable to effectively devote attentional resources to both the storage of information and maintenance of fluent writing processes. The fluency of an individuals’ writing (e.g., as measured by speed of writing) appears to play a role in determining their capacity to recall information (Peverly, 2006). Peverly found that the fluency of participants writing was correlated with how well they recalled information, with fluent writers able to recall more information from WM than dysfluent writers. However, in Peverly’s study, the data regarding fluency was gathered from one task and the data regarding recall was from a separate task. As such, it is unknown whether this pattern holds up when writing and WM tasks are completed simultaneously. To enable comparisons with previous studies that look at writing fluency and its effect on recall (e.g., Peverly, 2006), we will provide statistics for the mean kinematics of the writing movements (average stroke duration, average stroke size, and average absolute velocity). Further to this, we will investigate whether a relationship exists between the fluency of writing and number of words recalled from a concurrent WM task.

The above research proposes that both reading and writing may overload WM processes resulting in poor storage and retrieval of information while listening places minimal strain on WM. However, previous studies have failed to investigate whether writing, reading, and listening place different levels of strain on WM and if any of them places significantly more strain than the others. Specific to the current study, Bourdin and Fayol (1994, 2002) found that participants recalled fewer words in a serial recall task when they wrote down words compared to if they verbalised their responses by saying them aloud. While this supports other findings on the complexity of writing, it fails to determine if writing during encoding (i.e., when the words were initially presented) overloads WM’s ability to immediately recall information more than reading or listening during encoding. Writing appears to be more cognitively complex than reading and listening. We would therefore expect it to overload WM to a greater extent. However, this proposal has so far not been investigated.

To investigate whether reading, writing, and listening impact on recall to different degrees the current study asked participants to complete a serial recall task after they read, listened, and wrote down lists of words. It is expected that serial recall will differ between all three conditions, with recall being best in the listening condition, moderate in the reading condition and poorest in the writing condition. Further post hoc analysis will be conducted for the serial position curve to investigate whether this pattern holds between all the conditions at individual serial positions. This will provide more fine-grained information about how the WM processes are affected by the tasks.

As we will be employing a serial recall task it is likely that mistakes will occur in the form of order errors (Acheson & MacDonald, 2009; Henson, 1998)—that is, when an item is recalled correctly but in the incorrect serial position (Gathercole, 2008). Order errors will therefore be reported as they provide insight into how each task is affecting the underlying processes of WM (Acheson & MacDonald, 2009). For example, if the writing condition produces a higher proportion of order errors, this would provide evidence that the secondary writing task is preventing accurate phonological encoding (Acheson & MacDonald, 2009).

## Method

### Participants

Sixteen university students participated in this experiment. After checking for outliers, one of these participants was rejected from further analysis. This left 15 participants, seven male and eight female; with a mean age of 34.67 years (*SD* = 12.45). All participants were required to have normal or corrected to normal vision and hearing with English as their first language. Participants provided informed consent and the study was approved by the Southern Cross University Human Research Ethics Committee.

### Design

The experiment employed a repeated measures design. There were two independent variables: experimental condition, with three levels (reading, listening, and writing), and serial position, with six levels (position 1 to 6). The dependent variables were the proportion of words recalled, and the proportion of order errors. Participant recall was measured by correct responses following strict serial recall criteria (Acheson, Postle, & MacDonald, 2010; Conway et al., 2005). That is, a correct response was recorded if a word was recalled in the correct serial position. Mean accuracy for each serial position for each participant was used for further analysis. Order errors were analysed as the proportion of errors individuals made per condition. This was calculated by dividing the total number of order errors by the number of words recalled correctly in any position (Miller & Roodenrys, 2012).

### Apparatus

The experiment was conducted in a lab with a personal computer (screen resolution, 1,920×1,080) and a Wacom (Intuos3, 12”×19”, model PTZ-1231W) digitizer and stylus to record the words written by the participants. MovAlyzeR (Neuroscript LLC, USA) displayed the writing on a computer screen and recorded the pen movements. The participants used headphones to listen to a pre-recorded list of words spoken by the researcher, presented via E-prime on a second computer.

Three hundred and fifty words were obtained from the MRC psycholinguistics database (Coltheart, 1981). The parameters set for the words were the amount of letters (4-8), syllables (2), word familiarity (300-500), concreteness (200-500), and the Kucera and Francis (1967) frequency scale (1-75). The words were randomised using excel and the first 270 words were chosen. The words were portioned into three blocks of 15 lists, with six words per list. All three conditions contained every word block, the word blocks were organised and counterbalanced ensuring the same word block was not presented in the same experimental condition. The counterbalancing of the condition/block order was then randomised so every version of the presentation had an equal chance of being used. The conditions were counterbalanced so no participant had the same combination of word lists/condition. As the experiment was repeated measures, participants completed all three conditions.

The general structure of each condition was the same (i.e., words were presented then recalled). What changed was the task the participants performed. At the start of each condition a “START” icon appeared. To start a list of words participants needed to make a single stroke movement through the “START” icon using the stylus. This would trigger the beginning of a word list, between hitting the “START” icon and the presentation of the first word was a 1.5 s gap, then the first word was presented (auditory or visual). All the words were recorded by the researcher using Audacity and the files were saved as .wav files. The mean duration (presentation) of each individual word in the listening and writing condition was 916 ms (*SD* = 102 ms). The words in the reading condition were presented for 1 s before disappearing from the screen. For each condition, the next five words were presented at 3 s intervals, measured from the beginning of each word presentation. After the last word had been presented, there was a final 3 s gap and a beep to signal to the participants to stop the task they were doing. The total length of each word list was 18 s, after which the screen was cleared of any writing to prevent participants from gaining feedback to aid in recall. At this point, recall was prompted by a new screen that had “RECALL” written on the centre of the screen. This lasted for 30 s before a new “START” icon was displayed until the participant was ready to continue (participants could continue to recall after the 30 s if they needed to).

Recall was recorded using a response sheet with positions one to six. During the recall phase of the experiment participants were required to write down their responses by filling in the spaces provided with the word that corresponded with that serial position. Kinematic measures were calculated for each movement stroke. A stroke is the movement between points of zero velocity or local minima of absolute velocity. The writing kinematics were used as measures of writing fluency and calculated by the average stroke duration—that is, the average duration of a single movement (stroke) in seconds; average stroke size—that is, the average size of a single stroke (cm); and average absolute velocity—that is, the speed of movement (cm/s).

### Procedure

Participants completed the experiment individually. Upon entering the room, participants were greeted by the experimenter and were asked to take a seat in the cubicle where the experiment was to take place. Participants were asked to read the information sheet that outlined the purpose of the experiment, what would be required of them as well as instructing them of their rights to participate and withdraw. Participants then completed a consent form, which was signed and dated. The participants were then instructed on how the program worked and what they needed to do during the experiment.

Once this was completed, the experimenter instructed the participants that they were going to complete a WM task. Firstly, the participants were asked to place the headphones on and then a practice example was opened on MovAlyzeR and the experimenter walked the participant through the procedure. Participants were told they were going to complete a serial recall task. They were instructed that they would hear a list of words (listening and writing conditions) or read a list of words (reading condition). At the end of each list of six words there was a screen displaying “Recall”. Once this appeared, participants were told to recall the word lists by writing them down on the response sheet provided. They were given the example, if you were to hear/read the words *dog*, *cat*, *bat*, *elephant*, *rabbit*, and *spider* you are to recall them by writing them down in the same order you just heard or read, in the blank spaces provided. If you do not remember a word in a certain position, you are to leave that space “BLANK”. For example, if you do not remember the third word, you are to write, “Dog, cat, ______, elephant, rabbit, and spider”. Participants were told that each list had exactly six words and that there were fifteen lists in each condition. The procedure for recall was the same for all conditions.

Next, the participants were shown the “START” icon and told that throughout the experiment to start a new list they must move the stylus through the “START” icon. After doing this, they would hear/read a new list of words. Participants practiced before the start of each condition until they and the experimenter were comfortable with the program. At this point, the experimenter started the experimental condition and left the room. Participants were asked to contact the experimenter once the condition had been completed (this would be noticeable as the program shut after completion).

When completing the listening task, participants were asked to focus on an “X” that appeared at the centre of the screen after placing a stroke through the “START” icon (to control for individual variability during the task). Once the word list had ended, the word “RECALL” appeared on the screen and participants were told that this was where they were to begin recalling the words in the order they were presented. It was emphasised that all they needed to do was listen to the word lists and once recall appeared on the screen to begin to write down their responses on the provided response sheet.

During the reading condition participants were instructed to silently read the words that appeared on the screen in front of them after hitting the “START” icon. Participants continued to read until the word “RECALL” appeared at the centre of the screen; this was to prompt them to stop and begin to write down their responses on the response sheet.

In the writing condition, participants were instructed to move the stylus through the “START” icon to initiate each trial. They were then instructed to write down the words they were listening to, beginning immediately as the first word was presented. Participants were told to write in their natural writing and attempt to write down each word even if they were unsure of the correct spelling. It was emphasised that they must attempt to write down each word. Participants continued writing until the end of the list (i.e., 3 s after the final word was initially presented). At this point, the screen went blank and “RECALL” appeared on the centre of the screen. Participants could then begin to recall the words on the provided response sheet.

## Results

Recall was compared for all three experimental conditions (reading, writing, and listening) and serial positions through a repeated measures Analysis of Variance (ANOVA). The proportion of order errors were analysed with a repeated measures ANOVA to identify if there were significant differences between conditions. The descriptive statistics for the average number of words recalled (out of six) in the listening, reading, and writing conditions are provided in Table 1.

**Table 1.** Mean Number and Standard Deviation (*SD*) of Words Recalled per List in the Listening, Reading, and Writing Conditions

	Mean	*SD*
Listening	3.76	1.04
Reading	3.51	1.06
Writing	2.82	0.74

### Word Recall

Shapiro-Wilk’s and *F*_max_ analysis was used to test the assumptions of normality and homogeneity of variance, respectively. Shapiro-Wilk’s was not met for four variables, recall at serial position one in the reading condition, position one and six in the listening condition and position six in the writing condition. As there were few deviations from normality, they were considered not to be of concern (Allen & Bennett, 2010). *F*_max_ was not violated and homogeneity was assumed.

**Figure 1.**
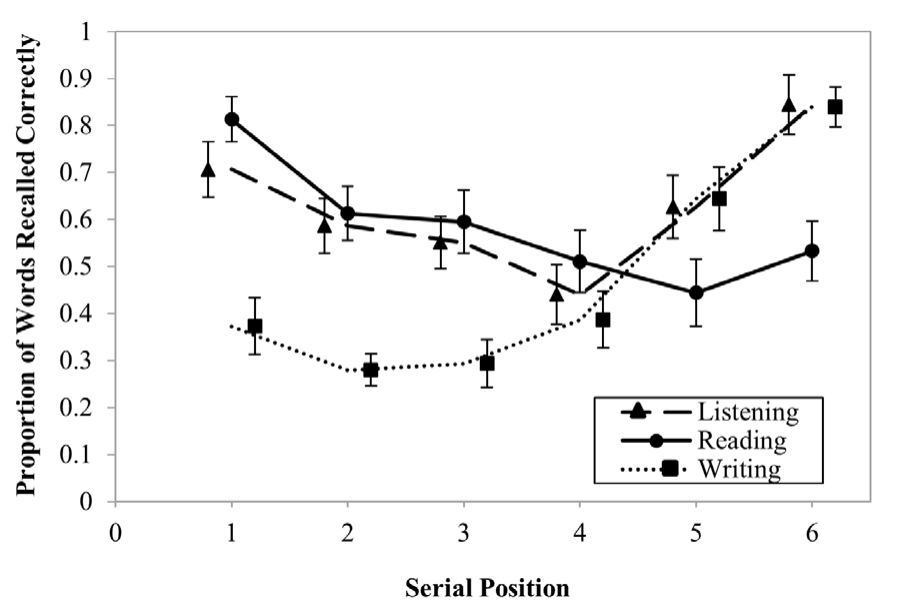
Mean word recall at each serial position as a proportion of correct responses for participants during the listening, reading, and writing conditions. Error bars represent standard errors. Points are offset horizontally so that error bars are visible.

Mauchley’s test was significant for serial item recall indicating assumptions of sphericity were not met, thus the Huynh-Feldt adjusted analysis was employed. Mauchley’s test was non-significant for the experimental conditions indicating assumptions of sphericity were met. Figure 1 summarises the mean words recalled in the writing, reading, and listening conditions at each serial position. On average, participants recalled fewer words in the writing condition at serial positions one to three compared to the reading and listening conditions. However, recall in the reading condition at serial positions five and six was less than the writing and listening conditions.

The repeated measures ANOVA revealed a main effect for experimental condition, *F*(2, 28) = 14.59, *p* < .001, ƞ^2^_p_ = .51 and serial position, *F*(1.90, 26.56) = 8.16, *p* = .002, ƞ^2^_p_ = .38. Bonferroni post hoc comparisons revealed that participants recalled significantly fewer words overall in the writing condition compared to the reading, *M*_Diff_ = .115, Bonferroni 95% CI [.19, .04], and listening conditions, *M*_Diff_ = .156, Bonferroni 95% CI [.24, .07]. There was no significant difference between the reading and listening conditions.

The ANOVA also revealed a significant interaction between experimental condition and serial position, *F*(10, 140) = 20.62, *p* < .001, ƞ^2^_p_ = .60. Further post hoc comparisons were conducted to determine at which serial position the differences occurred. A series of linear contrasts revealed a significant difference between the writing condition and the listening condition at serial positions one, *M*_Diff_ = .33, Bonferroni 95% CI [.50, .17], two, *M*_Diff_ = .31, Bonferroni 95% CI: [.44, .18], and three, *M*_Diff_ = .26, Bonferroni 95% CI [.41 ,.1]. This pattern was repeated for comparisons between the writing and reading conditions. Recall was worse for the writing condition at serial positions one, *M*_Diff_= .44, Bonferroni 95% CI [.56, .32], two, *M*_Diff_= .33, Bonferroni 95% CI [.47, .20], and three, *M*_Diff_ = .30, Bonferroni 95% CI [.48, .12].

A significant reduction in item recall for the reading condition compared to writing occurred at serial positions five, *M*_Diff_ = .20, Bonferroni 95% CI [.36, .04], and six, *M*_Diff_ = .31, Bonferroni 95% CI [.44, .17]. A reduction in the proportion of items recalled between the writing and the listening conditions was found at serial positions five, *M*_Diff_= .18, Bonferroni 95% CI [.29, .08], and six, *M*_Diff_ = .31, Bonferroni 95% CI [.48, .15]. There was no significant difference between reading and listening conditions at serial positions one, two, or three, or between the listening and writing conditions at serial positions five and six. There were no significant differences between any conditions at serial position four.

### Order errors

**Figure 2.**
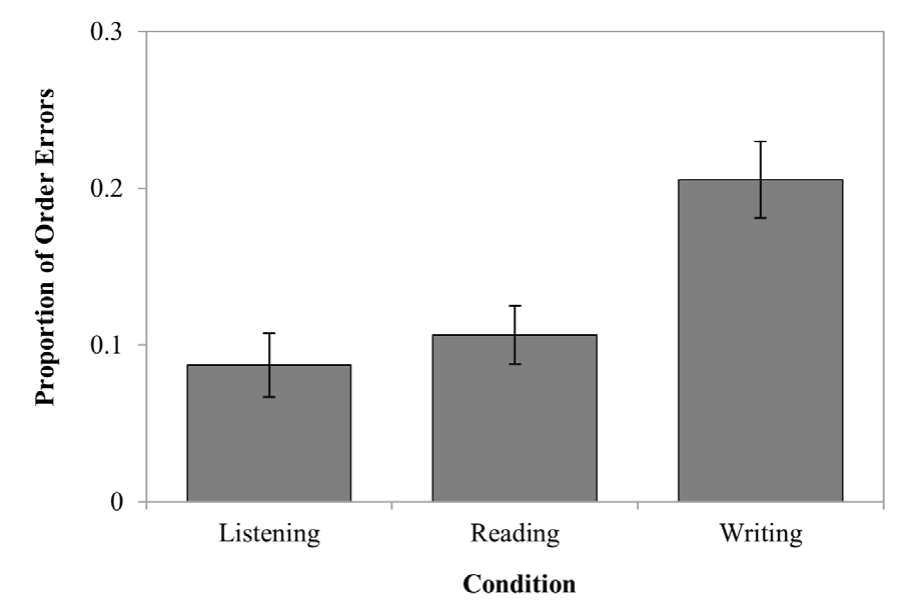
The mean proportion of order errors for the listening, reading, and writing conditions. Error bars represent the standard error of the mean.

Order errors were analysed as the proportion of errors individuals made per condition. Figure 2 summarises the mean proportion of order errors in the writing, reading, and listening conditions. The repeated measures ANOVA revealed a significant difference in the proportion of order errors between conditions, *F*(2, 28) = 13.51, *p* < .001, ƞ^2^_p_ = .49. Bonferroni post hoc comparisons revealed that there were significantly more order errors in the writing condition compared to the listening condition, *M*_Diff_= .12, Bonferroni 95% CI [.04, .2], and the reading condition, *M*_Diff_ = .10, Bonferroni 95% CI [.03, .17]. The listening and reading conditions did not differ.

### Writing Fluency

Descriptive statistics for writing fluency as measured by the average stroke duration, average stroke size, and average absolute velocity are reported in Table 2. Table 3 displays the results from a bivariate Pearson correlation for the proportion of words recalled in the writing condition and the kinematic measures of writing fluency. None of the correlations between the proportion of words recalled and kinematic measures of writing fluency reached significance. This indicates that no significant relationship exists between kinematic measures of writing fluency and performance on a concurrent WM task.

**Table 2.** Descriptive Statistics for the Measures of Writing Fluency

Kinematic	Mean	*SD*
Duration (ms)	116.51	66.75
Size (cm)	1.06	0.77
Absolute velocity (cm/s)	10.72	5.82

**Table 3.** Bivariate Pearson Correlations Between the Proportion of Words Recalled and the Mean Stroke Duration, Size and Absolute Velocity in the Writing Condition

		Duation	Size	Absolute velocity
Recall	*r*	.282	-.324	-.380
	*p*	.309	.239	.162

*Note*. *r* - Pearson correlation coefficient, *p* - obtained probability

## Discussion

This experiment investigated how the relative complexity of listening, reading, and writing during encoding of verbal information affects WM performance on a serial recall task. The results suggest that the writing process overloaded WM significantly more than just reading or listening when trying to encode words in memory. This pattern was also found between conditions at individual serial positions. However, differences only occurred at serial positions one to three between writing and both reading and listening. At position four, there was no difference, and at position five and six, there was no difference between writing and listening, but a significant difference between writing and reading, with more words recalled in the writing condition. This finding supports previous literature indicating that the writing process is cognitively complex. The results did not show a relationship between the kinematic measures of writing and serial recall performance. Therefore, the relationship observed by Peverly (2006) between a WM and writing task when performed independently of one another does not hold up when the two tasks are performed simultaneously. However, due to the small sample size a lack of power could have prevented the detection of a relationship between the two variables. Taken together, the results support the hypothesis that writing overloads WM and reduces recall performance compared to a listening and reading task.

The above results could be explained by the capacity of WM that is available for the recall task. The current study suggests listening places significantly less strain on WM (is less complex) than writing. This is consistent with previous research on the simplicity of listening tasks (Margolin et al., 1982) and the processing of phonologically coded material in WM (Baddeley, 2001). The listening condition also displayed a typical primacy and recency effect. As such, listening does not overload WM more than reading and writing. It appears that during the listening task participants were able to recall more words as they were able to devote more cognitive resources to sub-vocal rehearsal, as information was phonologically coded and could be directly stored in the phonological loop. Additionally, during the listening task, participants could direct attention towards processing and maintenance because no secondary process was being performed (Barrouillet & Camos, 2007). This allowed attention to be sustained on the to-be-remembered items without the need to inhibit distractors (Chun, 2011).

There was no difference for overall recall between the reading and listening tasks. However, the expected difference did occur at serial positions five and six, with more words recalled in the listening task. Our results demonstrate a typical primacy effect and a weaker recency effect for reading. The difference between the reading condition compared to the listening and writing conditions at these serial positions provides evidence that there are differences in the processing of the two types of verbal information (i.e., written and auditory). The results suggest that the processing of words in the reading task is less effective at the later stages of serial recall than listening and writing tasks. This could be due to a reduction in the efficiency of transforming the most recently presented items into a phonological code then storing and rehearsing them in memory before immediate recall begins. Conversely, we can see that auditory words (i.e., the listening and writing conditions) are being processed automatically as they are phonologically coded (Baddeley & Larsen, 2007; Haenggi & Perfetti, 1992). Further to this, the pattern of order errors proposes that the reading condition allowed efficient encoding of words (with no difference in order errors compared to listening). This suggests that the underlying processes involved in reading do not disrupt WM as much as a concurrent writing task.

Based on overall recall, the most cognitively complex task is writing, where there is the additional process of converting the phonological information to its written form and programming and performing the writing movements (Kellogg, 1996). The design of the experiment is such that the only difference between the listening and writing tasks was writing words as the participants listened to them. This takes up more of the limited WM resources, leaving less available for encoding and storage (Barrouillet & Camos, 2007; Chun, 2011). Previous research has indicated that the writing process utilises WM (Benton, Kraft, Glover, & Plake, 1984; Kellogg, 1996; McCutchen, 2000; Olive, 2004; Tse et al., 2014), which explains why writing places significantly more strain on WM than reading and listening.

The observed pattern for recall in the writing condition suggests some level of interference during the encoding, rehearsal, or maintenance of words while the words are being written. The rehearsal/maintenance of the first items presented is inhibited by the writing during encoding and recall. The ability to recall the most recently presented items is indistinguishable between the writing and listening conditions, possibly because the concurrent writing pauses once the last word has been presented. This could allow the participants directing their attention towards maintaining the most recently presented items before recall begins (Barrouillet & Camos, 2007).

The results in our experiment show that writing is more cognitively demanding as identified by the reduction in recall and increase in order errors compared to the reading and listening conditions. The increase in order errors implies that the words are not being encoded efficiently within the phonological loop while writing (Acheson & MacDonald, 2009), as resources are divided between processes (Barrouillet & Camos, 2007). This prevents items from being stored and retrieved at the correct serial position (Acheson & MacDonald, 2009). Conversely, no differences in order errors occurred between the listening and reading conditions, which corresponds to the main effect for recall in the repeated measures analysis. This reinforces that writing is a cognitively complex and demanding process that disrupts encoding and prevents accurate recall of to-be-remembered words, compared to reading and listening.

The increase in recall at serial position five and six in the writing condition is consistent with what is expected for serial recall of verbally presented stimuli (Hurlstone, Hitch, & Baddeley, 2014; Logie, Della Sala, Wynn, & Baddeley, 2000; Saito, Logie, Morita, & Law, 2008; Tan & Ward, 2008). We argue that the writing condition did not elicit poorer recall at these serial positions as the methodology allowed immediate recall. As shown in previous serial recall tasks of verbally presented stimuli (Pattamadilok, Lafontaine, Morais, & Kolinsky, 2010; Saito et al., 2008; Spurgeon, Ward, & Matthews, 2014), the recency effect is due to short term memory’s ability to hold those items relatively well and sustain them for immediate recall.

In light of this, our findings can be interpreted as follows. The writing pauses after the presentation of the final word, allowing attentional resources to be switched back to encoding and maintenance. The items can therefore be rehearsed/maintained much more efficiently. In contrast, after encoding each of the early words, the writing task continues as participants write down subsequent words, leading to a weaker memory trace for the earlier words. The memory trace for the most recently presented item is stronger as it remains activated in short term memory for immediate retrieval (Pattamadilok et al., 2010). While this explanation is consistent with our results, further research will be required for confirmation. The novel effect of poor recall during the beginning of the serial position list (in the writing condition) is worth being investigated further and may help explain the cognitive effects of writing on WM.

## Conclusion

The current study fills a void in the literature demonstrating that writing overloads WM more than reading and listening, leading to worse recall of concurrently presented words. This indicates that writing is more cognitively complex and places a greater strain on WM processes than reading and listening. The cognitive requirements associated with writing (Kellogg, 1996) could be preventing attention from switching back to processing and maintaining items within WM (Barrouillet & Camos, 2007). Further to this, we have identified some of the ways the writing and reading processes interfere with WM processes, as revealed in the pattern across serial recall. The results suggest that a trade-off exists between task complexity, and retaining information in WM. That is, the more complex a task or the more difficult it is to perform by an individual, the fewer words are recalled in a concurrent verbal WM task. Furthermore, this has a differential impact on earlier or later words in a list depending on the WM processes affected. On a practical note, these findings have implications for situations such as lectures and meetings where there is a requirement that information is retained during and immediately following its presentation. We suggest that under these circumstances, writing while listening will not lead to the highest degree of overall recall and that it may be better to simply pay attention and listen.

## References

[R1] Acheson D. J., MacDonald M. C. (2009). Verbal working memory and language production: Common approaches
to the serial ordering of verbal information.. Psychological Bulletin.

[R2] Acheson D. J., Postle B. R., MacDonald M. C. (2010). The interaction of concreteness and phonological similarity in
verbal working memory.. Journal of Experimental Psychology: Learning, Memory, and
Cognition.

[R3] Allen P. J., Bennett K. (2010). PASW statistics by SPSS: A practical guide: Version 18.0..

[R4] Baddeley A. (1997). Human memory: Theory and practice..

[R5] Baddeley A. (2000). The episodic buffer: A new component of working
memory?. Trends in Cognitive Sciences.

[R6] Baddeley A. (2001). Is working memory still working?. American Psychologist.

[R7] Baddeley A. (2003). Working memory: Looking back and looking forward.. Nature Reviews Neuroscience.

[R8] Baddeley A. (2012). Working memory: Theories, models, and
controversies.. Annual Review of Psychology.

[R9] Baddeley A, Hitch G. (1974). Working memory. In G. Bower (Ed.), The psychology of learning and
motivation: Advances in research and theory (Vol. 8, pp. 47-90)..

[R10] Baddeley A., Larsen J. D. (2007). The phonological loop: Some answers and some
questions.. Quarterly Journal of Experimental Psychology.

[R11] Barrouillet P., Camos V., Osaka N., Logie R. H., D’Esposito M. (2007). The time-based resource-sharing model of working
memory.. The cognitive neuroscience of working memory.

[R12] Benton S. L., Kraft R. G., Glover J. A., Plake B. S. (1984). Cognitive capacity differences among writers.. Journal of Educational Psychology.

[R13] Besner D., Davelaar E. (1982). Basic processes in reading: Two phonological
codes.. Revue Canadienne de Psychologie.

[R14] Bourdin B., Fayol M. (1994). Is written language production more difficult than oral language
production? A working memory approach.. International Journal of Psychology.

[R15] Bourdin B., Fayol M. (2002). Even in adults, written production is still more costly than oral
production.. International Journal of Psychology.

[R16] Chen Z., Cowan N. (2009). Core verbal working-memory capacity: The limit in words retained
without covert articulation.. Quarterly Journal of Experimental Psychology.

[R17] Christensen T. A., Almryde K. R., Fidler L. J., Lockwood J. L., Antonucci S. M., Plante E. (2012). Modulating the focus of attention for spoken words at encoding
affects frontoparietal activation for incidental verbal
memory.. International Journal of Biomedical Imaging.

[R18] Chun M. M. (2011). Visual working memory as visual attention sustained internally
over time.. Neuropsychologia.

[R19] Coltheart M. (1981). The MRC psycholinguistic database.. Quarterly Journal of Experimental Psychology.

[R20] Conway A. R. A., Kane M. J., Bunting M. F., Hambrick D. Z., Wilhelm O., Engle R. W. (2005). Working memory span tasks: A methodological review and
user’s guide.. Psychonomic Bulletin & Review.

[R21] Davidson B. J. (1986). Activation of semantic and phonological codes during
reading.. Journal of Experimental Psychology: Learning, Memory, and
Cognition.

[R22] Folk J. R. (1999). Phonological codes are used to access the lexicon during silent
reading.. Journal of Experimental Psychology: Learning, Memory, and
Cognition.

[R23] Gathercole S. (2008). Working memory. In J. Byrne (Series Ed.) & H. L. Roediger, III (Vol.
Ed.), Learning and memory: A comprehensive reference: Vol. 2 Cognitive
psychology of memory (pp. 33-52)..

[R24] Haenggi D., Perfetti C. A. (1992). Individual differences in reprocessing of text.. Journal of Educational Psychology.

[R25] Henson R. N. (1998). Short-term memory for serial order: The start-end
model.. Cognitive Psychology.

[R26] Hurlstone M. J., Hitch G. J., Baddeley A. D. (2014). Memory for serial order across domains: An overview of the
literature and directions for future research.. Psychological Bulletin.

[R27] Kellogg R. T., Levy C. M., Ransdell S. E. (1996). A model of working memory in writing.. The science of writing. Theories, methods, individual differences and
applications.

[R28] Kellogg R. T. (2001). Presentation modality and mode of recall in verbal false
memory.. Journal of Experimental Psychology: Learning, Memory, and
Cognition.

[R29] Klein K., Boals A. (2001). Expressive writing can increase working memory
capacity.. Journal of Experimental Psychology: General.

[R30] Kucera H., Francis W. N. (1967). Computational analysis of present-day American English..

[R31] Lewandowsky S., Farrell S., Nadel L. (2006). Computational models of working memory. Encyclopedia of cognitive science.

[R32] Linderholm T., Xiaosi C., Qin Z. (2008). Differences in low and high working-memory capacity
readers’ cognitive and metacognitive processing patterns as a
function of reading for different purposes.. Reading Psychology.

[R33] Logie R. H., Della Sala S., Wynn V., Baddeley A. (2000). Visual similarity effects in immediate verbal serial
recall.. The Quarterly Journal of Experimental Psychology Section A, Human
Experimental Psychology.

[R34] Margolin C. M., Griebel B., Wolford G. (1982). Effect of distraction on reading versus
listening.. Journal of Experimental Psychology: Learning, Memory, and
Cognition.

[R35] McCutchen D. (1996). A capacity theory of writing: Working memory in
composition.. Educational Psychology Review.

[R36] McCutchen D. (2000). Knowledge, processing, and working memory: Implications for a
theory of writing.. Educational Psychologist.

[R37] Miller L. M., Roodenrys S. (2012). Serial recall, word frequency, and mixed lists: The influence of
item arrangement.. Journal of Experimental Psychology: Learning, Memory, and
Cognition.

[R38] Oberauer K., Lewandowsky S. (2008). Forgetting in immediate serial recall: Decay, temporal
distinctiveness, or interference?. Psychological Review.

[R39] Olive T. (2004). Working memory in writing: Empirical evidence from the dual-task
technique.. European Psychologist.

[R40] Page M. P. A., Norris D. (1998). The primacy model: A new model of immediate serial
recall.. Psychological Review.

[R41] Pattamadilok C., Lafontaine H., Morais J., Kolinsky R. (2010). Auditory word serial recall benefits from orthographic
dissimilarity.. Language and Speech.

[R42] Peverly S. T. (2006). The importance of handwriting speed in adult
writing.. Developmental Neuropsychology.

[R43] Rayner K., Pollatsek A., Ashby J., Clifton C. Jr. (2012). Psychology of reading..

[R44] Sadoski M., Paivio A., Ruddell R. B., Unrau N. J. (2004). A dual coding theoretical model of reading.. Theoretical models and processes of reading (5th ed.).

[R45] Saito S., Logie R. H., Morita A., Law A. (2008). Visual and phonological similarity effects in verbal immediate
serial recall: A test with kanji materials.. Journal of Memory and Language.

[R46] Schweppe J., Rummer R. (2013). Attention, working memory, and long-term memory in multimedia
learning: An integrated perspective based on process models of working
memory.. Educational Psychology Review.

[R47] Spurgeon J., Ward G., Matthews W. J. (2014). Examining the relationship between immediate serial recall and
immediate free recall: Common effects of phonological loop variables but
only limited evidence for the phonological loop. Journal of Experimental
Psychology.. Learning, Memory, and Cognition.

[R48] Tan L., Ward G. (2008). Rehearsal in immediate serial recall.. Psychonomic Bulletin & Review.

[R49] Tirre W. C., Peńa C. M. (1992). Investigation of functional working memory in the reading span
test.. Journal of Educational Psychology.

[R50] Tse L. F. L., Thanapalan K. C., Chan C. C. H. (2014). Visual-perceptual-kinesthetic inputs on influencing writing
performances in children with handwriting difficulties.. Research in Developmental Disabilities.

[R51] Unsworth N., Engle R. W. (2007). The nature of individual differences in working memory capacity:
Active maintenance in primary memory and controlled search from secondary
memory.. Psychological Review.

